# Inhibition of the AT-Hook DNA-Binding Domain Attenuates HMGA2-Mediated Epithelial Mesenchyme Transition in Esophageal Cancer Cells

**DOI:** 10.3390/ijms27156933

**Published:** 2026-08-02

**Authors:** Lucas de Jesus Lima, Matheus Lohan-Codeço, Maria Luísa Barambo Wagner, Isabella Paiva Ramos de Oliveira, Arthur Renato Macedo Adade, Luiz Marcelo Ribeiro Tomé, Nathalia Meireles Da Costa, Luís Felipe Ribeiro Pinto, Luiz Eurico Nasciutti, Mariana Severo Ramundo, Antonio Palumbo

**Affiliations:** 1 Laboratório de Interações Celulares, Instituto de Ciências Biomédicas, Programa de Pesquisa em Biologia Celular e do Desenvolvimento, Universidade Federal do Rio de Janeiro, Prédio do Centro de Ciências da Saúde—Cidade Universitária, Ilha do Fundão, Rua César Pernetta, 1766 (LS.3.01), Rio de Janeiro 21941-902, Brazil; lucasdjlima2@gmail.com (L.d.J.L.); marilubw12@gmail.com (M.L.B.W.); isabellapschool@gmail.com (I.P.R.d.O.);; 2 Programa de Carcinogênese Molecular (PCM), Centro de Pesquisa e Inovação (CPQI), Instituto Nacional de Câncer (INCA), Rua André Cavalcanti 37, Centro, Rio de Janeiro 20231-050, Brazil; nathalia.meireles@inca.gov.br (N.M.D.C.); frpinto@inca.gov.br (L.F.R.P.); 3 Departamento de Clínica Médica, Disciplina de Imunologia Clínica e Alergia, Faculdade de Medicina da Universidade de São Paulo, São Paulo 01246-903, Brazil; 4 Laboratório de Imunologia, LIM-19, Instituto do Coração (INCOR), Hospital das Clínicas HCFMUSP, Faculdade de Medicina da Universidade de São Paulo, São Paulo 05403-900, Brazil

**Keywords:** esophageal squamous cell carcinoma, HMGA proteins, netropsin, epithelial–mesenchymal transition, AT-rich DNA sites

## Abstract

Esophageal squamous cell carcinoma (ESCC) is a highly prevalent malignancy worldwide. Moreover, ESCC remains poorly characterized at the molecular level, which contributes to limited therapeutic options and an overall poor prognosis. In this context, HMGA family members, which are overexpressed in tumors but almost absent in healthy adult tissues, seem to represent promising therapeutic targets. These proteins act by binding to AT-hook DNA-binding motifs and may regulate the expression of several genes associated with tumor progression. Therefore, integrating in silico, translational, and in vitro approaches, we investigated the functional consequences of blocking HMGA2–DNA interaction in ESCC tumor progression by using netropsin, a site-specific ligand for AT-rich DNA regions. Our results demonstrate that netropsin treatment significantly reduced cell viability, migration, and cell cycle progression, thereby promoting apoptosis. Furthermore, netropsin treatment was capable of partially reverting Epithelial–Mesenchymal Transition (EMT) activation associated with HMGA2 expression, by downregulating EMT activators, such as Slug and Twist. Finally, the netropsin treatment sensitizes ESCC cells to chemotherapeutic treatment with 5-Fluorouracil. Taken together, our findings highlight that AT binding-specific blockade could be correlated with the inhibition of HMGA2 and may reveal a promising approach to better understand ESCC progression.

## 1. Introduction

Esophageal cancer (EC) is a highly aggressive malignancy characterized by elevated incidence and mortality, ranking as the 11th most common cancer and the 7th leading cause of cancer-related death worldwide [1]. It comprises two major histopathological subtypes, Esophageal Squamous Cell Carcinoma (ESCC) and Esophageal Adenocarcinoma (EAC), which differ in their geographic distribution, incidence, risk factors, and underlying pathogenesis [2]. ESCC accounts for approximately 90% of all esophageal cancer cases, with its principal risk factors including excessive alcohol consumption, tobacco smoking, the habitual intake of very hot beverages, and diets deficient in micronutrients [3,4]. Because ESCC is frequently diagnosed at advanced stages, it is associated with an unfavorable prognosis [5,6]. Despite recent advances in the understanding and management of this disease, further studies are required to elucidate the molecular mechanisms underlying esophageal carcinogenesis and to identify biomarkers for early detection, as well as novel therapeutic targets that may contribute to the development of more effective treatment strategies and ultimately improve patient outcomes. In this context, the High Mobility Group A (HMGA) family comprises three architectural chromatin-binding proteins—HMGA1, HMGA1b, and HMGA2—which are characterized by their ability to bind adenine- and thymine-rich DNA sequences (AT-rich regions) through their AT-hook domains within the minor groove of DNA [7]. Aberrant overexpression of these proteins has been implicated in the initiation and progression of multiple tumor types by altering chromatin architecture and indirectly regulating the transcription of numerous genes [8,9,10,11]. Specifically, in ESCC, HMGA2 overexpression has been reported in approximately 90% of tumor specimens, and its silencing markedly reduces cellular phenotypes associated with tumor progression [12], suggesting that functional inhibition of HMGA2 may represent a promising therapeutic strategy for this malignancy. In this context, netropsin is a naturally occurring cationic oligopeptide isolated from *Streptomyces netropsis*. Structurally, it consists of two N-methylpyrrole rings linked by amide bonds and exhibits high affinity for AT-rich DNA sequences within the minor groove, thereby preventing members of the HMGA protein family from binding to these regions [13], making it a promising antagonist of HMGA proteins. Notably, Miao and collaborators demonstrated that netropsin is capable of inhibiting HMGA2 activity with high specificity [14]. Given the established role of HMGA2 in the biology of several malignancies, including ESCC [12], the present study aimed to investigate the functional consequences of pharmacologically disrupting HMGA2 binding to DNA in an ESCC model.

## 2. Results

### 2.1. Netropsin Treatment Decreases the Malignant Progression of ESCC Cells

**Figure 1:**
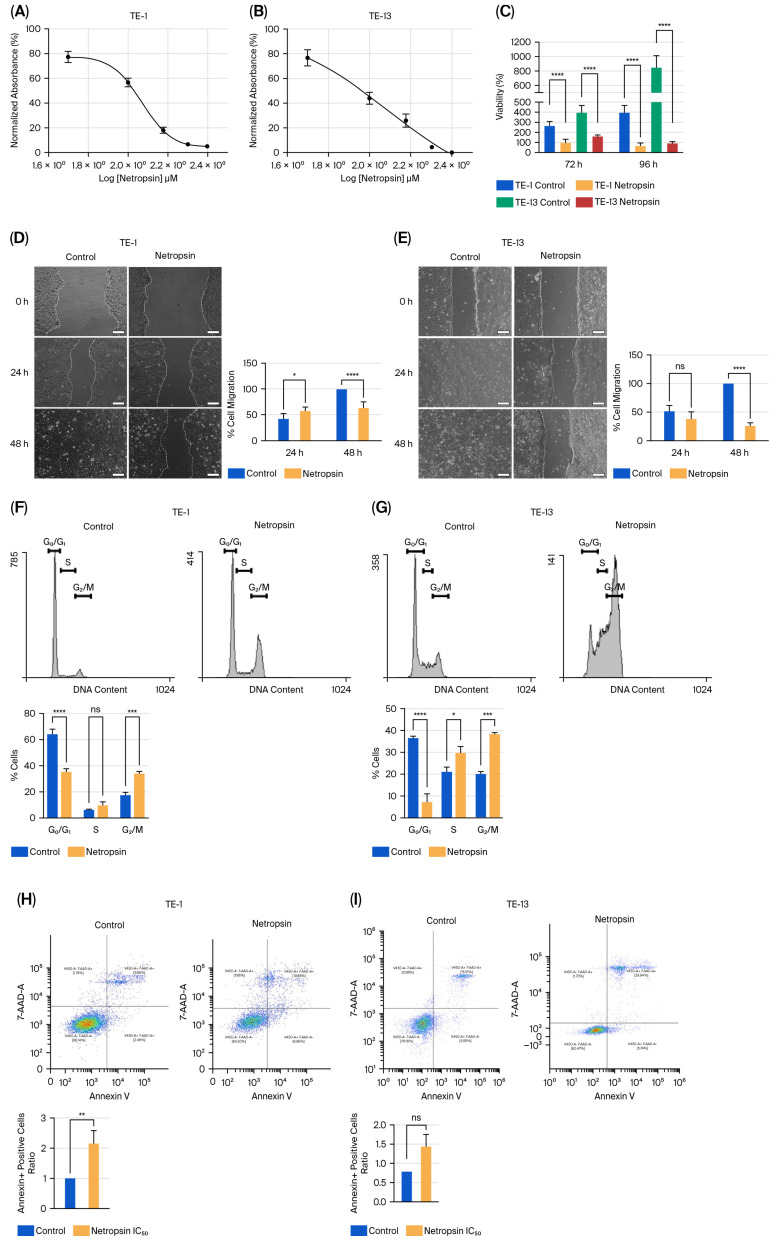
Netropsin treatment impacts important cellular parameters associated with ESCC in vitro progression. (**A**,**B**) Dose–response curves evaluating cell viability (nor-malized absorbance) of TE-1 (**A**) and TE-13 (**B**) cell lines after treatment with different concentrations of netropsin (50, 100, 150, 200, and 250 µM) for 72 h to determine the IC_50_. (**C**) Cell viability of ESCC cell lines TE-1 and TE-13 after treatment with netropsin determined IC_50_ for 72 h and 96 h. (**D**,**E**) Migration assay (wound healing) in TE-1 (**D**) and TE-13 (**E**) cells at 0 h, 24 h, and 48 h after treatment with netropsin determined IC_50_.Images are representative, and the quantification refers to the migration percentage over time. Scale bar = 300 µm for all images. (**F**,**G**) Cell cycle distribution analysis in TE-1 (**F**) and TE-13 (**G**) cell lines after 72 h of treatment with netropsin determined IC_50_. (**H**,**I**) Assessment of apoptotic cell death (Annexin-V/7-AAD) in ESCC cell lines TE-1 (**H**) and TE-13 (**I**). Bar graphs quantify the ratio of Annexin-V-positive cells. All data are represented as mean ± standard deviation (SD) of three independent experiments. * *p* < 0.05; ** *p* < 0.01; *** *p* < 0.001; **** *p* < 0.0001; ns = non-significant.

After determining the IC_50_ values for the ESCC cell lines TE-1 (117.4 µM) and TE-13 (120.6 µM) (Figure 1A,B), functional assays were performed to investigate whether netropsin could antagonize malignant phenotypes classically associated with HMGA2 expression. Netropsin treatment significantly reduced both cell viability (Figure 1C) and migratory capacity (Figure 1D) in the two ESCC cell lines. Moreover, it increased the proportion of cells in the S and G2/M phases of the cell cycle (Figure 1F,G) in both cell lines and significantly elevated the percentage of Annexin V-positive cells (Figure 1H) compared with their respective control groups. Collectively, these findings indicate that netropsin impairs cell cycle progression and promotes apoptotic cell death in ESCC cells. These findings are consistent with the well-established effects of DNA-targeting agents, which activate the DNA damage response (DDR), induce S-phase and G2/M cell-cycle arrest, and, when DNA lesions cannot be efficiently repaired, trigger apoptotic cell death through sustained checkpoint activation, mitotic failure, and the inability to re-enter the G1 phase [15].

### 2.2. HMGA2 Expression Is Associated with EMT Pathway Activation in ESCC

**Figure 2:**
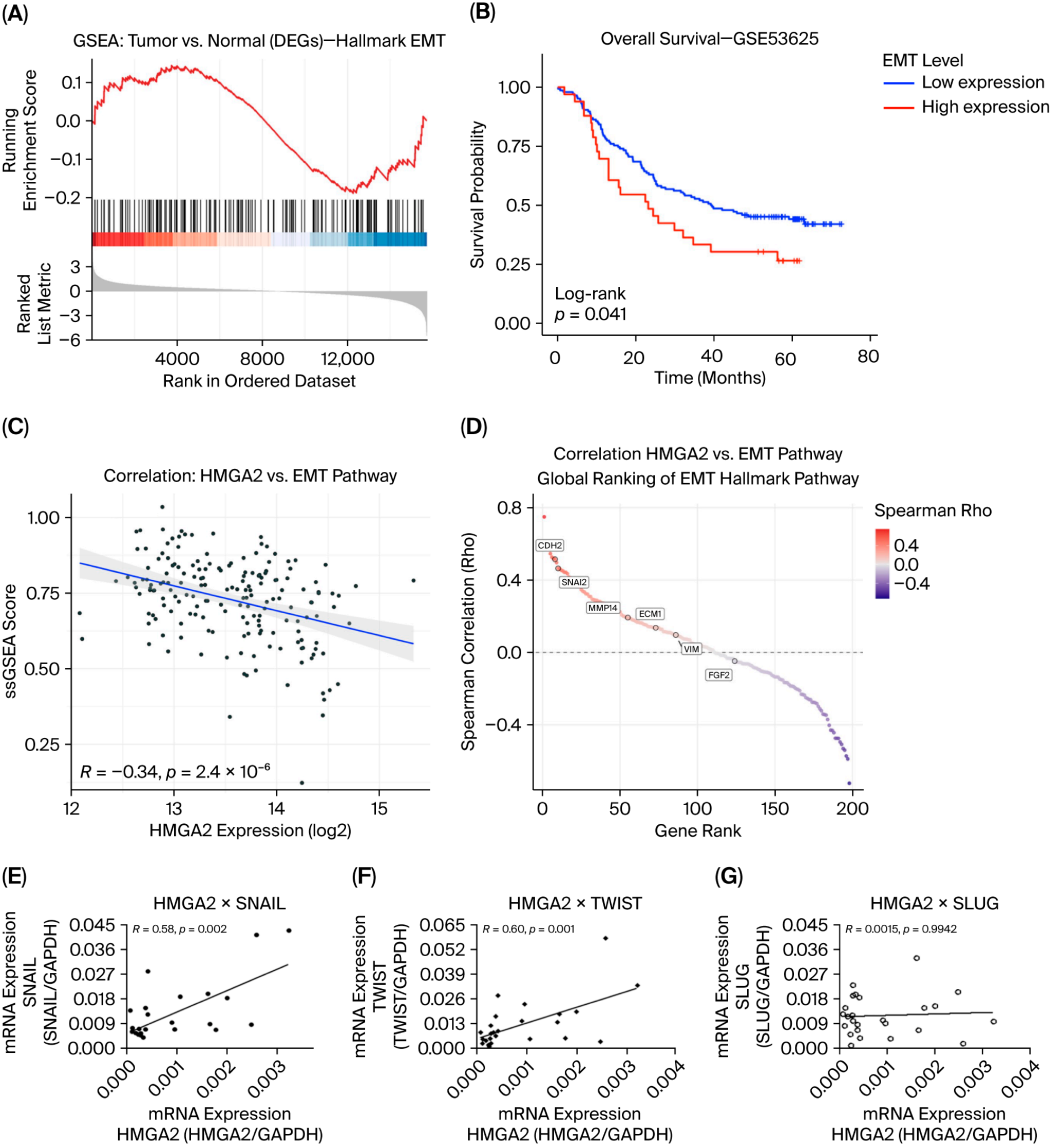
*HMGA2* expression associates with the Epithelial–Mesenchymal Transition (EMT) pathway. (**A**) Gene Set Enrichment Analysis (GSEA) for the Hallmark EMT signature, comparing expression profiles of tumor samples and normal tissue. (**B**) Overall survival curve (Kaplan–Meier) of patients from the GSE53625 cohort, stratified by high (red) or low (blue) EMT pathway expression. (**C**) Scatter plot demonstrating the correlation between the single-sample enrichment score (ssGSEA) for the EMT pathway and *HMGA2* expression levels (log2). (**D**) Overall ranking of the Spearman correlation (Rho) between *HMGA2* expression and individual genes of the Hallmark EMT signature, highlighting structural and regulatory markers (*CDH2*, *SNAI2* and *VIM*). (**E**–**G**) Validation of the correlation of relative mRNA expression (normalized by that of *GAPDH*) between *HMGA2* and EMT-inducing transcription factors: Snail (**E**), Twist (**F**), and Slug (**G**) in tumor samples from patients with ESCC. Gene correlation analyses were evaluated with the respective coefficients (R or Rho) indicated in the panels. Differences in survival probability were calculated using the log-rank statistical test (*p* = 0.041).

Since netropsin antagonized malignant phenotypes classically associated with HMGA2 expression, we next investigated, through in silico analyses, which HMGA2-regulated pathways and biological processes in ESCC could potentially be affected by this compound. To this end, we performed differential gene expression analysis followed by Gene Set Enrichment Analysis (GSEA) using the GEO dataset GSE53625, which identified the Epithelial–Mesenchymal Transition (EMT) pathway as one of the most significantly enriched biological programs (Figure 2A). To determine whether EMT pathway activation correlates with poor clinical outcomes, we used the MSigDB Hallmark EMT gene set to establish an EMT signature and applied single-sample gene set enrichment analysis (ssGSEA) to assess EMT-related gene expression in each sample. Based on these scores, survival analysis demonstrated that ESCC patients displaying high expression of EMT signature genes (Figure 2B) exhibited poorer overall survival. To further investigate the relationship between *HMGA2* expression and the EMT pathway, a correlation analysis was performed using *HMGA2* expression levels and the previously generated ssGSEA EMT scores. This analysis revealed an overall inverse correlation between *HMGA2* expression and EMT pathway activity (Figure 2C). However, when EMT signature genes were analyzed individually, a distinct pattern emerged. Although *HMGA2* expression was not globally associated with EMT signature genes, its overexpression was positively correlated with key EMT regulators, including *CDH2*, *SNAI2*, and *VIM* (Figure 2D). Since in silico analyses demonstrated a strong association between *HMGA2* and the EMT pathway, the correlation between *HMGA2* expression and major EMT transcriptional regulators was further evaluated in ESCC patient samples. A significant correlation was observed between *HMGA2* and the genes encoding the transcription factors Snail and Twist (Figure 2E,F), but not Slug (Figure 2G).

### 2.3. Netropsin Treatment Partially Reverts the EMT Phenotype in ESCC Cells

**Figure 3:**
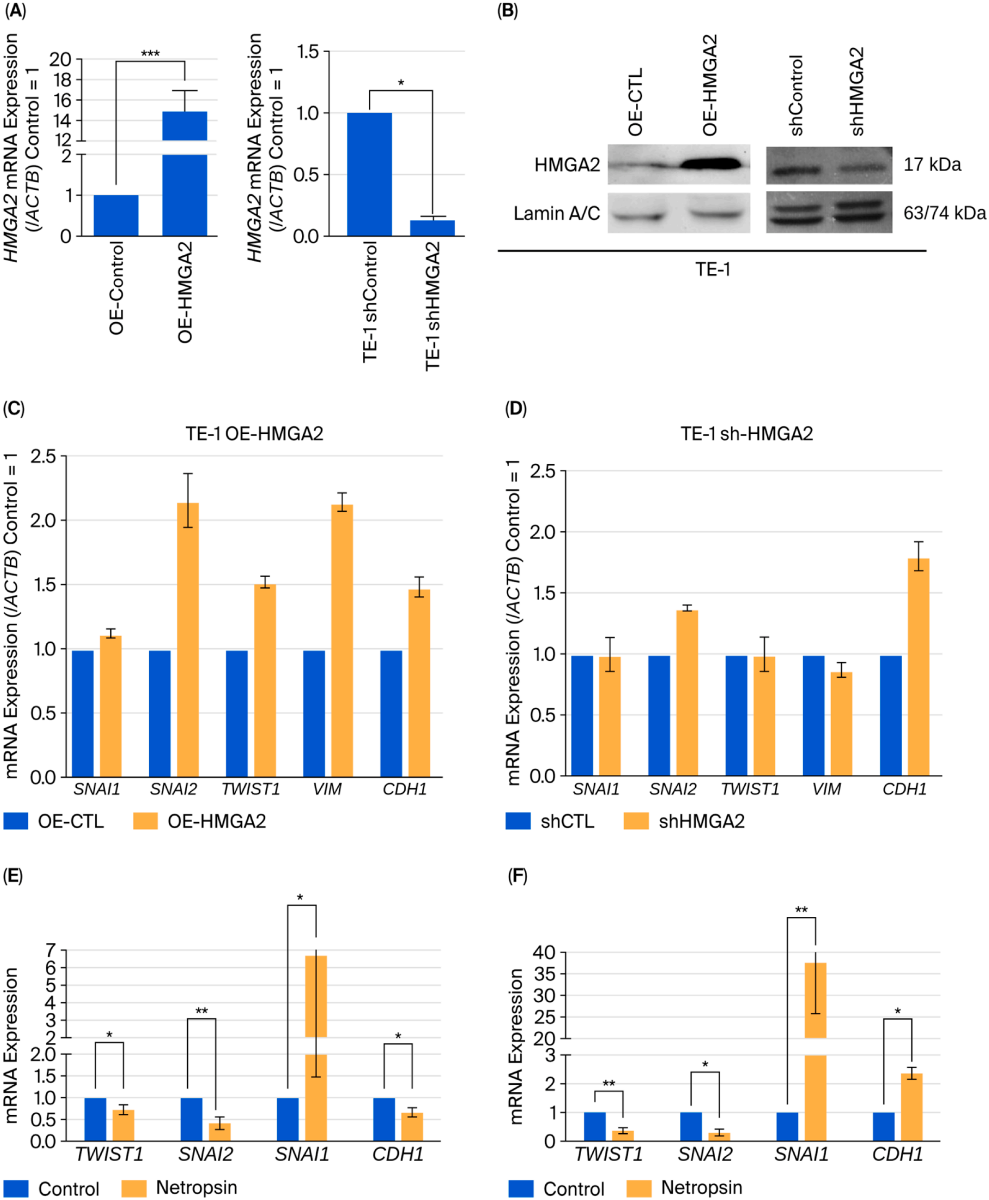
HMGA2 modulation and netropsin treatment reshape the expression of EMT-associated transcription factors in ESCC cell lines. (**A**) qPCR validation of HMGA2 modulation in TE-1 cells stably overexpressing HMGA2 (OE-HMGA2) or expressing an HMGA2-targeting shRNA (shHMGA2), relative to their respective controls. (**B**) Representative Western blotting images confirming HMGA2 protein overexpression and knockdown in TE-1 cells; Lamin A/C was used as a loading control. (**C**,**D**) qPCR analysis of EMT-associated markers *SNAI1*, *SNAI2*, *TWIST1*, *VIM* and *CDH1* in TE-1 cells upon HMGA2 overexpression (**C**) or silencing (**D**). (**E**,**F**) qPCR analysis of *TWIST1*, *SNAIL2*, *SNAIL1* and *CDH1* in TE-1 (**E**) and TE-13 (**F**) cells treated with IC_50_ of netropsin for 72 h. * *p* < 0.05; ** *p* < 0.01; *** *p* < 0.001; ns = non-significant.

**Figure 4:**
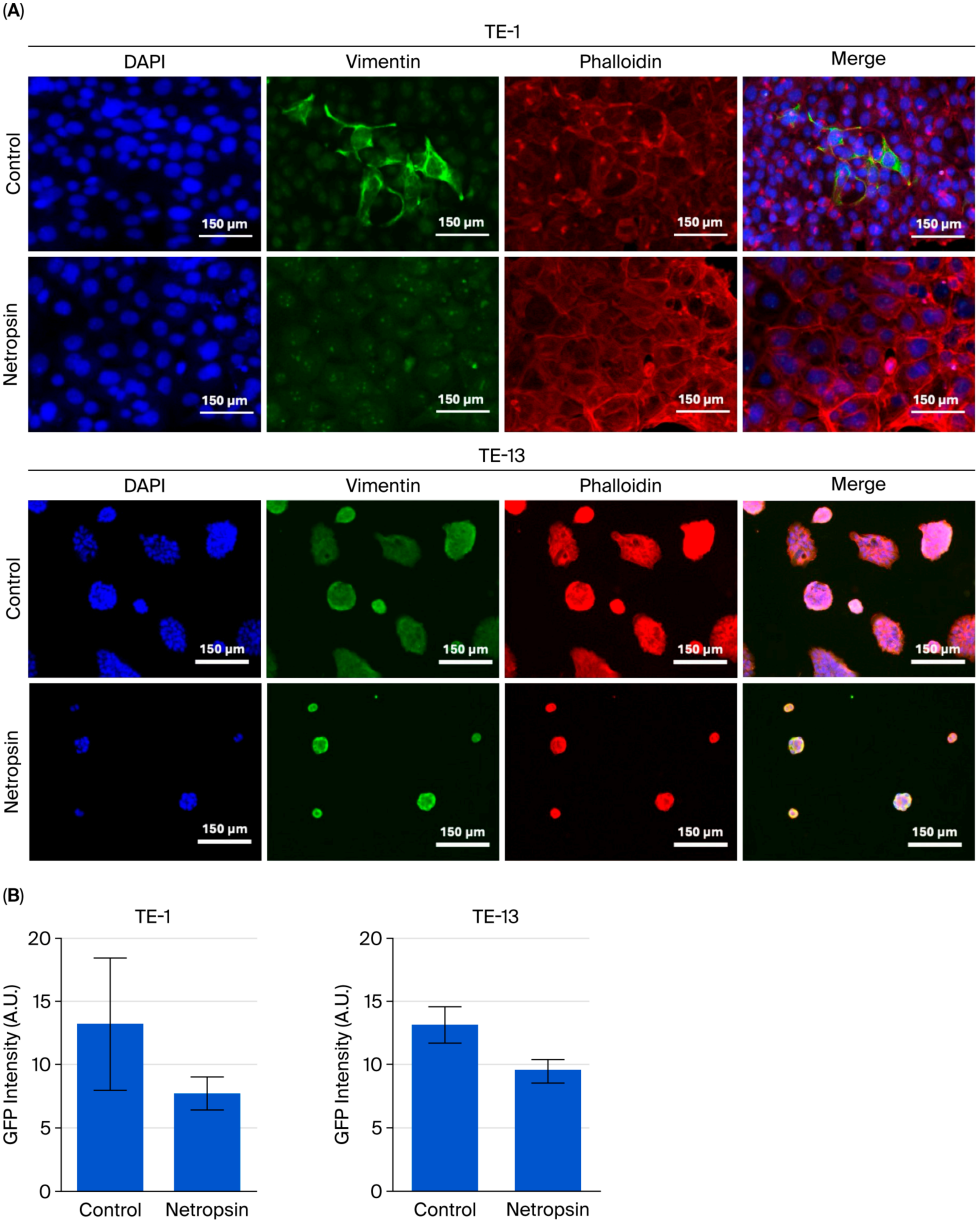
Netropsin reduces vimentin expression and remodels the actin cytoskeleton in ESCC cells. (**A**) Immunofluorescence evaluating protein expression and distribution of vimentin (green) in TE-1 and TE-13 ESCC cell lines cultured in the absence (top) and presence (bottom) of netropsin IC_50_ treatment for 72 h. Nuclei were contrasted with DAPI (blue), and the actin cytoskeleton was labeled with phalloidin (red). Images were acquired with a 20× objective using the EVOS M5000 Imaging System (Thermo Scientific). Scale bars: 150 µm. (**B**) Quantification of vimentin fluorescence intensity in TE-1 (**left**) or TE-13 (**right**) cells, expressed in arbitrary units (A.U.).

Based on the association identified between *HMGA2* expression and the EMT pathway in ESCC, we next investigated whether this molecular profile could be recapitulated in vitro through modulation of *HMGA2* expression. Therefore, since netropsin binds with high affinity to the minor groove of AT-rich DNA sequences, which are the same regions preferentially recognized by the AT-hook domains of HMGA2 and sterically hinders HMGA2 from interacting with its target DNA sequences. As a result, HMGA2 loses its ability to modulate chromatin architecture and regulate the transcription of downstream target genes [14]. After confirming the efficiency of stable *HMGA2* overexpression and knockdown (Figure 3A,B), we found that *HMGA2* overexpression markedly increased the expression of *SNAI2* and *TWIST1*, accompanied by a modest increase in *SNAI1*. Unexpectedly, *HMGA2* overexpression also resulted in elevated *CDH1* expression (Figure 3C). Conversely, *HMGA2* silencing reversed the expression pattern of EMT-associated transcription factors while also markedly increasing *CDH1* expression (Figure 3D). Collectively, these findings are consistent with our translational and in silico analyses and further support a close association between HMGA2 expression and EMT activation in ESCC. Next, we investigated whether netropsin treatment could modulate the expression of key transcription factors involved in EMT activation. Netropsin significantly reduced the expression of *TWIST1* and *SNAI2* while increasing *SNAI1* expression in both ESCC cell lines (Figure 3E,F). Consistent with the elevated *SNAI1* levels, *CDH1* expression was reduced in TE-1 cells (Figure 3E). In contrast, TE-13 cells exhibited the opposite response, showing increased *CDH1* expression following netropsin treatment (Figure 3F). Since netropsin modulated the expression of EMT-associated regulators, we next evaluated the mesenchymal marker vimentin [16] by immunofluorescence. In agreement with the gene expression findings, netropsin markedly reduced vimentin expression in both cell lines (Figure 4A,B). This effect was accompanied by reorganization and retraction of the actin cytoskeleton, particularly in TE-13 cells, as revealed by phalloidin staining (Figure 4A,B).

### 2.4. Netropsin Abrogates Malignant Behaviors Associated with EMT Pathway Activation

**Figure 5:**
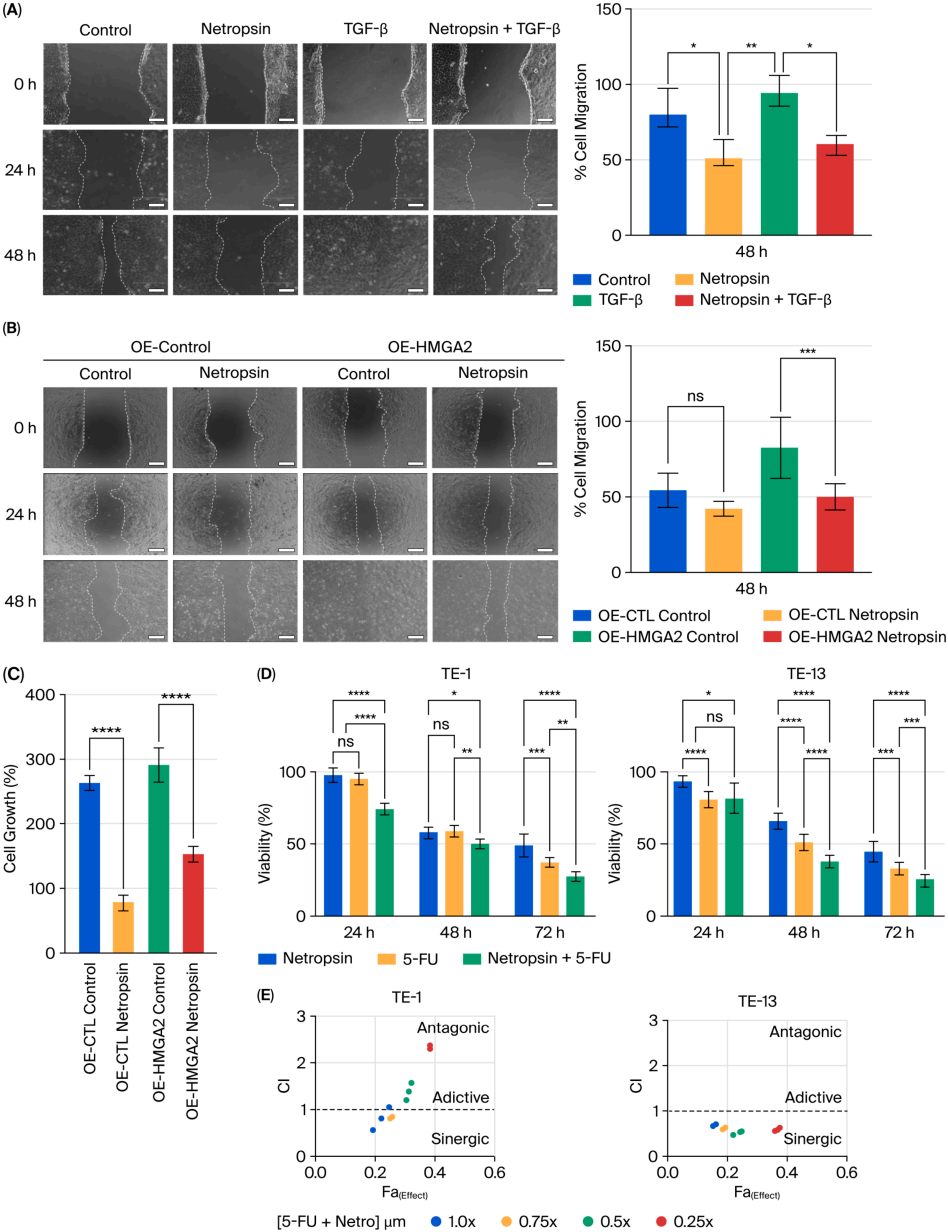
Netropsin reverses the TGF-β-induced migratory phenotype and exhibits synergism in 5-Fluorouracil (5-FU) sensitization. (**A**) Wound healing assay in TE-1 cells at 0 h, 24 h, and 48 h after treatment with the IC_50_ (117.4µM) of netropsin, or TGF-β (10 ng/mL), or the IC_50_ of netropsin + TGF-β (10 ng/mL). Images are representative, and quantification refers to the percentage of migration after 48 h. (**B**) Wound healing assay in TE-1 OE-Control and TE-1 OE-HMGA2 cells at 0 h, 24 h, and 48 h after treatment with the IC_50_ of netropsin. Images are representative, and quantification refers to the percentage of migration after 48 h. Scale bar = 300 µm. (**C**) Evaluation of cell growth of TE-1 cells (OE-Control and OE-HMGA2) after 96 h of culture in the presence or absence of netropsin. (**D**) Evaluation of cell viability in TE-1 and TE-13 cell lines over 72 h of culture in the presence of netropsin, or 5-Fluorouracil (5-FU) or netropsin + 5-FU. (**E**) Analysis of the pharmacological interaction between 5-FU and netropsin in TE-1 and TE-13 cell lines, using Combination Index (CI) plots based on the Chou-Talalay method. The dashed line (CI = 1) delimits the additive effect; CI values < 1 indicate synergism and CI > 1 indicate antagonism in the different effect fractions (Fa). The data represent the mean ± standard deviation (SD) of three independent replicates. * *p* < 0.05; ** *p* < 0.01; *** *p* < 0.001; **** *p* < 0.0001; ns = not significant.

Subsequently, we investigated whether the molecular changes induced by netropsin were accompanied by functional alterations associated with EMT activation in ESCC cells. Given that netropsin negatively modulated the expression of key EMT-related regulators, we evaluated its effects on TGF-β-induced cell migration. Accordingly, migration assays were performed in the presence of the canonical EMT inducer TGF-β, either alone or in combination with netropsin. As expected, TGF-β treatment significantly enhanced the migratory capacity of TE-1 cells (Figure 5A). Notably, co-treatment with netropsin markedly attenuated the promigratory effects induced by TGF-β (Figure 5A). Furthermore, given that HMGA2 is frequently overexpressed in ESCC and that netropsin inhibits HMGA2 binding to DNA, thereby blocking its transcriptional activity [17], we next investigated the effects of netropsin in ESCC cells stably overexpressing HMGA2. Consistent with the findings obtained in the parental cell lines, netropsin significantly inhibited both cell migration and proliferation in HMGA2-overexpressing ESCC cells (Figure 5B,C). Finally, these results demonstrate that netropsin effectively counteracts HMGA2-driven malignant phenotypes, particularly those associated with EMT activation in ESCC. In addition, given previous evidence that aberrant HMGA2 expression influences the response of colorectal cancer cells to 5-fluorouracil (5-FU) [18], we investigated whether netropsin could modulate the sensitivity of ESCC cells to 5-FU. To this end, ESCC cell lines were exposed to netropsin and 5-FU, either individually or in combination. Co-treatment resulted in a greater reduction in cell growth than either agent alone (Figure 5D). To determine whether this enhanced effect reflected additive or synergistic interactions, drug combination analyses were performed using the Chou–Talalay method [19] across the IC_50_ values of each compound and their respective fractional concentrations. Under these conditions, TE-1 cells exhibited synergistic interactions at the 1.0× and 0.75× IC_50_ fractions, whereas TE-13 cells displayed synergism across all concentrations evaluated (Figure 5E).

## 3. Discussion

Esophageal cancer remains one of the most lethal malignancies worldwide and is associated with a particularly poor prognosis [20]. HMGA2 is frequently overexpressed in ESCC [12] and represents an attractive therapeutic target due to its established role in tumor initiation and progression, as well as its association with aggressive disease and chemoresistance [21]. Indeed, several studies have shown that reducing HMGA2 expression attenuates malignant phenotypes across multiple tumor types [22,23]. Accordingly, pharmacological inhibition of HMGA2 has emerged as a promising therapeutic strategy, although its clinical potential remains to be fully explored [24,25,26,27]. In this study, we investigated the effects of netropsin, a DNA minor groove ligand that specifically binds adenine- and thymine-rich sequences, thereby preventing the interaction of HMGA proteins with their target DNA regions [24]. Using an in vitro model of ESCC, we demonstrated that netropsin significantly reduced key hallmarks of tumor progression, including cell viability, migration, and cell cycle progression, while promoting apoptotic cell death. Collectively, these findings are consistent with the well-established oncogenic functions of HMGA2, which promotes proliferation and cell cycle progression through multiple mechanisms [28,29], while also regulating signaling pathways involved in cell survival [30] and migration [31].

Furthermore, to gain mechanistic insights into the effects of HMGA2 inhibition following netropsin treatment, we performed in silico analyses that revealed a close association between HMGA2 expression and the EMT pathway in ESCC. When HMGA2 expression was correlated with the overall EMT signature, a weak inverse association was observed. However, gene-level analyses revealed a distinct pattern, with HMGA2 expression showing significant positive correlations with key EMT-associated genes, including *CDH2*, *VIM*, and *SNAI2*. To further validate these findings, we assessed the relationship between *HMGA2* expression and the major EMT-inducing transcription factors in an independent cohort of ESCC patient samples. *HMGA2* expression was positively correlated with *SNAI1* and *TWIST1*, whereas no significant association was detected with *SNAI2* (Slug). Together, these findings suggest that HMGA2 does not globally regulate the EMT transcriptional program in ESCC but rather promotes a selective EMT-related gene expression profile through the modulation of specific downstream targets. This interpretation is supported by previous studies demonstrating that HMGA2 promotes EMT through multiple signaling pathways, including TGF-β/SMAD and MAPK/ERK signaling [21]. In this context, it is important to emphasize that ESCC is a highly heterogeneous malignancy strongly influenced by distinct etiological factors, the prevalence of which varies considerably across geographic regions [2]. Accordingly, population-specific molecular characteristics should be taken into account when interpreting transcriptomic data. In the present study, the validation cohort consisted of Brazilian patients, whereas the ESCC cases included in TCGA are predominantly derived from Asian populations, particularly Chinese patients. Therefore, the apparent discrepancies observed in the correlation between HMGA2 expression and EMT-related regulators may, at least in part, reflect population-specific molecular differences. Supporting this hypothesis, previous studies have demonstrated that the TP53 mutational spectrum of Brazilian ESCC closely resembles that reported in French cohorts, while differing substantially from that observed in Chinese patients [32]. In this context, we investigated whether netropsin could modulate the expression of key transcription factors involved in EMT regulation in vitro. Our results demonstrated that netropsin significantly downregulated TWIST and SLUG, while also reducing the expression of the mesenchymal marker vimentin. Interestingly, however, *SNAIL1* expression was significantly increased in both ESCC cell lines. Upregulation of *SNAIL1* has been classically associated with the repression of E-cadherin expression during EMT [33], a pattern observed in TE-1 cells but not in TE-13 cells. Together, these findings suggest that pharmacological inhibition of HMGA2 by netropsin selectively modulates the EMT transcriptional program, attenuating several mesenchymal features while indicating that additional regulatory mechanisms may contribute to the differential control of E-cadherin expression in ESCC. In this regard, Tan et al. [34] demonstrated that HMGA2 promotes EMT through the coordinated regulation of SNAIL and TWIST. Importantly, the authors showed that these transcription factors exert partially redundant functions during EMT, such that activation of either factor alone is sufficient to sustain mesenchymal traits. These observations are partially consistent with our findings. In TE-13 cells, netropsin treatment induced a clear attenuation of the EMT phenotype, characterized by reduced TWIST and SLUG expression, decreased vimentin protein levels, and increased E-cadherin expression, despite the concomitant upregulation of *SNAIL1*. Interestingly, Tan et al. also demonstrated that TWIST suppression can influence SNAIL expression, suggesting that the increased *SNAIL1* levels observed following netropsin treatment may represent a compensatory response to the marked downregulation of TWIST. Consistent with this hypothesis, HMGA2 overexpression in our model markedly increased *TWIST* expression, accompanied by a modest increase in *SNAIL1*, whereas HMGA2 silencing reversed the expression of both transcription factors. Moreover, Tan et al. demonstrated that HMGA2 represses E-cadherin expression through mechanisms that are, at least in part, independent of SNAIL and TWIST, indicating that HMGA2 can regulate epithelial identity through additional molecular pathways. This observation may also help explain our findings in TE-1 cells. Although netropsin treatment reduced *TWIST* and SLUG expression while increasing *SNAIL1*, E-cadherin expression was modestly decreased rather than restored. Therefore, the reduction in E-cadherin expression observed in TE-1 cells may reflect either compensatory *SNAIL1* upregulation or the persistence of HMGA2-dependent regulatory mechanisms that operate independently of the canonical EMT transcription factors. Finally, these findings raise the possibility that the biological effects of netropsin are not exclusively mediated through HMGA2 inhibition. In addition to preventing HMGA2 binding to AT-rich DNA regions, netropsin has been reported to interact with other AT-rich DNA-binding proteins and regulatory elements [35]. Consequently, modulation of these additional targets may also contribute to the molecular and phenotypic changes observed in the present study. Additionally, these findings should be interpreted with caution, as the phenotype induced by netropsin appears to be compatible with a partial epithelial–mesenchymal transition (partial EMT) state [36]. Under these conditions, tumor cells acquire a hybrid epithelial–mesenchymal phenotype in which epithelial and mesenchymal transcriptional programs coexist, rather than being regulated in a strictly antagonistic manner [37]. Increasing evidence indicates that this intermediate cellular state is not merely a transitional phase but a stable and biologically relevant phenotype associated with enhanced cellular plasticity, collective migration, extracellular matrix remodeling, metastatic dissemination, and resistance to chemotherapy [38,39]. Therefore, the selective modulation of EMT markers observed following netropsin treatment may reflect a shift toward a partial EMT state rather than a complete reversal of the EMT program. Nevertheless, these findings reveal an apparently paradoxical pattern. Although *SNAIL1* expression was consistently increased in both ESCC cell lines following netropsin treatment, the distinct effects observed on E-cadherin expression suggest that, in addition to the mechanisms discussed above, the intrinsic molecular heterogeneity of ESCC should also be considered. Given that TE-1 and TE-13 cells harbor distinct genetic and epigenetic backgrounds, different regulatory networks are likely to govern E-cadherin expression in each model. Consequently, HMGA2 inhibition may engage context-dependent molecular mechanisms that differentially influence the epithelial transcriptional program.

Indeed, although *SNAIL1* directly binds to the *CDH1* promoter, efficient repression of E-cadherin transcription requires the recruitment of multiple transcriptional co-repressors and chromatin-remodeling complexes, including histone deacetylases (HDACs) [40]. Consequently, increased *SNAIL1* expression alone may not be sufficient to suppress *CDH1* transcription, particularly in cellular contexts in which these co-repressors are absent, functionally impaired, or differentially regulated. This context-dependent regulatory mechanism may therefore explain the distinct patterns of E-cadherin expression observed in TE-1 and TE-13 cells following netropsin treatment. Since HMGA2-mediated EMT has been shown to involve activation of the TGF-β signaling pathway [41], and HMGA2 overexpression promotes cell migration and invasion through EMT induction [31], we next investigated whether netropsin could counteract these promalignant effects. To this end, migration assays were performed in ESCC cells stimulated with TGF-β or stably overexpressing HMGA2. In both experimental settings, netropsin effectively suppressed the enhanced migratory phenotype induced by either TGF-β stimulation or HMGA2 overexpression. Moreover, netropsin significantly reduced cell viability even in HMGA2-overexpressing cells, further supporting its ability to antagonize HMGA2-driven oncogenic signaling. Finally, given the reported association between HMGA2 overexpression and reduced sensitivity to 5-fluorouracil (5-FU) [42], we investigated whether netropsin could enhance the therapeutic response of ESCC cells to this chemotherapeutic agent. Combined treatment with netropsin and 5-FU produced a greater inhibitory effect on cell growth than either agent alone. Moreover, drug interaction analyses revealed a synergistic effect between the two compounds, suggesting that pharmacological inhibition of HMGA2 may enhance the antitumor efficacy of 5-FU in ESCC.

In conclusion, the present study demonstrates the antitumor potential of pharmacological inhibition of HMGA2 activity by netropsin in ESCC, particularly through the modulation of EMT-associated phenotypes. Nevertheless, several limitations should be considered when evaluating the translational potential of this approach. Netropsin has well-recognized drawbacks, including limited target specificity and dose-limiting toxicity [43]. Moreover, HMGA2 exerts biological functions that extend beyond its interaction with AT-rich DNA sequences, including protein–protein interactions and chromatin remodeling [29], which are unlikely to be completely abolished by preventing its DNA binding alone. These considerations should therefore be taken into account when interpreting the findings of the present study. Despite these limitations, our results demonstrate that pharmacological blockade of AT-rich DNA regions by netropsin attenuates multiple malignant phenotypes associated with HMGA2 overexpression in ESCC, particularly those related to EMT activation. These findings provide further evidence supporting HMGA2 as a therapeutically actionable target in ESCC and reinforce the rationale for the development of next-generation HMGA2 inhibitors with improved specificity and safety profiles. Given that aberrant HMGA2 expression is largely restricted to malignant tissues [44,45], targeting this protein represents a particularly attractive strategy for selective anticancer intervention.

## 4. Materials and Methods

### 4.1. Cell Lines

The TE-1 (CVCL_1759) and TE-13 (CVCL_4463) cell lines, derived from ESCC, were kindly provided by Dr. Pierre Hainaut (IARC, Lyon, France). The cells were cultured in RPMI-1640 medium (Gibco, Thermo Fisher Scientific, Waltham, MA, USA) supplemented with 10% fetal bovine serum (Gibco), 1% L-glutamine (Invitrogen, Thermo Fisher Scientific, Waltham, MA, USA), and 1% penicillin/streptomycin (Invitrogen), and maintained at 37 °C in a humidified atmosphere containing 5% CO_2_. The TE-1 OE-CTRL, TE-1 OE-HMGA2, TE-1 sh-Control and TE-1 sh-HMGA2 cell lines were established by our group transfecting vectors ORF-Control, ORF-HMGA2, sh-Control and sh-HMGA2 (GeneCopoeia, Rockville, MD, USA) using Lipofectamine 3000 (Invitrogen—L3000015) according to the manufacturer’s instructions and cultured under the same conditions described above, with the addition of 0.2% Geneticin (G418—Sigma-Aldrich, St. Louis, MO, USA) for stably HMGA2 overexpression cell lines and 0.5 µg/mL of puromycin (P8833—Sigma-Aldrich) for stably silenced HMGA2 cell lines.

### 4.2. Netropsin IC_50_ Determination and Cell Viability

To determine the Inhibitory Concentration of 50% (IC_50_) for netropsin (Sigma-Aldrich—N9653), TE-1 and TE-13 cell lines were seeded at a density of 5 × 10^3^ cells per well in 96-well plates and treated with increasing concentrations of netropsin (50, 100, 150, 200, and 250 µM). Seventy-two hours after treatment initiation, the culture medium was removed, and 200 µL of 3-(4,5-dimethyl-2-thiazolyl)-2,5-diphenyl-2H-tetrazolium bromide (MTT—Sigma-Aldrich) solution at 125 µg/mL, previously diluted in culture medium, was added and incubated at 37 °C for 3 h. Subsequently, absorbance was measured at 595 nm. To evaluate cell viability following combined treatment with 5-fluorouracil (5-FU, Fauldflour, Libbs Farmacêutica, São Paulo, Brazil) and netropsin, TE-1 and TE-13 cell lines were seeded in 96-well plates at a density of 5 × 10^3^ cells per well. Twenty-four hours after seeding, cells were treated with the IC_50_ concentration of netropsin and 5-FU for 72 h and then subjected to a viability assay using CCK-8 (TargetMol, Boston, MA, USA), according to the manufacturer’s instructions.

### 4.3. Cell Migration (Wound Healing Assay)

Cells were seeded in 24-well plates at a density of 1 × 10^5^ cells per well and allowed to grow until reaching 90–100% confluence. Subsequently, cells were treated with 10 µg/mL mitomycin C (Sigma Aldrich—M4287), diluted in culture medium, for 2 h. After treatment, the culture medium was removed, cells were washed with PBS, and a scratch was created across the cell monolayer using a 200 µL pipette tip. Cells were then incubated with either control culture medium or culture medium supplemented with the IC_50_ concentration of netropsin or 10 ng/mL of TGF-β (PeproTech, Thermo Fisher Scientific, Cranbury, NJ, USA; 100-21C). Images were acquired at 0 h, 24 h, and 48 h, and wound closure was quantified using the EVOS M5000 Imaging System (Thermo Fisher Scientific).

### 4.4. Cell Cycle Analysis

In 6-well plates, 2 × 10^5^ cells were seeded for cell cycle analysis. Twenty-four hours after seeding, cells were treated with either control culture medium or culture medium supplemented with the IC_50_ concentration of netropsin. After 72 h, cells were harvested and fixed for staining with propidium iodide (Invitrogen, F10797) according to the manufacturer’s protocol. Cell cycle distribution was analyzed using a flow cytometer (FACSCalibur, BD Biosciences, San Jose, CA, USA) and subsequently processed using Flowing Software 2.5.1 (Perttu Terho, Turku Bioscience Centre, University of Turku, Turku, Finland).

### 4.5. Apoptosis Assay

To evaluate apoptosis, 2.5 × 10^5^ cells per well were seeded in 6-well plates and allowed to adhere for 24 h. Cells were then treated with either control culture medium or culture medium supplemented with the IC_50_ concentration of netropsin. Seventy-two hours after treatment initiation, cells and supernatant were collected and stained with Annexin V and 7-AAD according to the manufacturer’s instructions (eBioscience Annexin V Apoptosis Kit; Invitrogen, Thermo Fisher Scientific, San Diego, CA, USA). The percentage of Annexin V–positive cells was determined by using the web-based software Floreada.io (https://floreada.io, accessed on 24 February 2025).

### 4.6. Synergism Between 5-Fluorouracil and Netropsin

The interaction between netropsin and 5-FU was evaluated using the Chou–Talalay method [19]. Briefly, cells were treated for 72 h with concentrations corresponding to 0.25×, 0.50×, 0.75×, and 1.0× of the IC_50_ of each drug, alone or in combination. Cell viability was assessed using the MTT assay, as previously described. The resulting data were analyzed using CompuSyn software version 1.0 (ComboSyn Inc., Paramus, NJ, USA) to generate isobolograms. Drug interactions were quantified based on the combination index (CI) as a function of the fraction affected (Fa), where CI = 1 indicates an additive effect, CI > 1 indicates antagonism, and CI < 1 indicates synergism.

### 4.7. RNA Extraction, Reverse Transcription, and qPCR

Total RNA from ESCC cell lines was extracted using the TRIzol reagent (Invitrogen), according to the manufacturer’s instructions. For reverse transcription, 750 ng of total RNA was converted into cDNA using the GoScript kit (Promega, Madison, WI, USA), and mRNA expression levels were detected by qPCR using the QuantiNova SYBR Green PCR Kit (Qiagen, Hilden, Germany) with gene-specific primers. GAPDH or β-Actin was used as an endogenous control for qPCR normalization. Relative gene expression was calculated using the 2^^−ΔΔC^ method. qPCR primer sequences follow:

*GAPDH* Sense: 5′-CAACAGCCTCAAGATCATCAGCAA-3′ and Antisense: 5′-AGTGATGGCATGGACTGTGGTCA-3′; *ACTB* Sense: 5′-CGCCAACACAGTGCTGTCT-3′ and Antisense: 5′-CACGGAGTACTTGCGCTCAG-3′ *SNAI1* Sense: 5′-AATACTGCAACAAGGAATACCTCAGCCT-3′ and Antisense: 5′-GGACAGGAGAAGGGCTTCTCGCCAGTG-3′; *SNAI2* Sense: 5′-CTTCCTGGTCAAGAAGCA-3′ and Antisense: 5′-GGGAAATAATCACTGTATGTGTG-3′; *TWIST1* Sense: 5′-TGTCCGCGTCCCACTAGC-3′ and Antisense: 5′-TGTCCATTTTCTCCTTCTCTGGA-3′;*CDH1* Sense: 5′-GAATGACAACAAGCCCGAAT-3′ and Antisense: 5′-GACCTCCATCACAGAGGTTCC-3′; *HMGA2* Sense: 5′-GCGCCTCAGAAGAGAGGAC-3′ and Antisense: 5′-GGTCTCTTAGGAGAGGGCTCA-3′.

### 4.8. Immunofluorescence

Cells grown on sterile glass coverslips were fixed with 4% paraformaldehyde for 10 min at room temperature, washed with PBS, and subjected to antigen retrieval with NH_4_Cl (50 mM) for 30 min. Cells were then permeabilized with 0.2% Triton™ X-100 (Sigma-Aldrich, X100) for 5 min and blocked with 5% BSA for 30 min. Primary antibody against vimentin (working dilution 1:50, Molecular Probes—Invitrogen—V2258) was diluted in PBS containing 3% BSA and incubated overnight at 4 °C in a humidified chamber. After washing, cells were incubated with FITC-conjugated secondary antibodies (1:100; Alexa Fluor^®^ 488, Molecular Probes—Invitrogen—A11001) for 2 h at room temperature, protected from light.

F-actin was stained with ActinRed 555 ReadyProbes Reagent (Invitrogen—R37112) for 30 min. After washing, nuclei were stained with DAPI (Invitrogen, D1306) for 20 min, and coverslips were mounted using N-propyl gallate mounting medium. Fluorescence images were acquired using the EVOS M5000 Imaging System (Thermo Fisher Scientific, Waltham, MA, USA).

Image quantification was performed in Image J version 1.54 (National Institutes of Health, Bethesda, MD, USA). Images were converted to 8-bit, and the mean fluorescence intensity of the vimentin channel was measured for each field. Values are expressed in arbitrary units (A.U.)

### 4.9. Western Blotting

Stably modulated HMGA2 cell lines were seeded at 2.5 × 10^5^ cells/well in 6-well plates. After 48 h, cells were harvested and lysed for 30 min in RIPA-like buffer (50 mM Tris-HCl pH 7.4, 250 mM NaCl, 0.1% SDS, 2 mM DTT, 0.5% NP-40) supplemented with Complete Mini EDTA-free protease inhibitor (Roche Diagnostics, Mannheim, Germany; 4693159001). Protein concentration was determined by Bradford assay (Bio-Rad Laboratories, Hercules, CA, USA; #500-0006) against a spectrophotometric standard curve. Thirty micrograms of protein were mixed with Laemmli buffer (200 mM Tris-HCl pH 6.8, 8% SDS, 40% glycerol, 0.01% bromophenol blue, 10% β-mercaptoethanol), denatured at 95 °C for 5 min, resolved by SDS-PAGE, and transferred onto nitrocellulose membranes using the iBlot Dry Blotting System (Invitrogen).

Membranes were blocked for 1 h in TBS-T (0.1% Tween-20) containing 5% non-fat dry milk and incubated overnight at 4 °C with anti-HMGA2 (rabbit, 1:1000; Invitrogen, PA5-21320) or anti-Lamin A/C (mouse, 1:1000; Cell Signaling Technology, Danvers, MA, USA; 4777S). Then, membranes were incubated for 2 h at room temperature with HRP-conjugated anti-rabbit (goat, 1:5000; Cell Signaling Technology, 7074S) or anti-mouse (goat, 1:5000; Cell Signaling, 7076) secondary antibodies. Following six 10 min washes, signals were detected by chemiluminescence (ECL Western Blotting Substrate, Promega, W1001).

### 4.10. In Silico Analyses

Gene expression data from patients with ESCC were obtained from the Gene Expression Omnibus (GEO) database under accession code GSE53625. Differential gene expression (DGE) analysis of microarray data was conducted using the limma package (version 3.66.0) in the R environment (R version 4.5.3; R Foundation for Statistical Computing, Vienna, Austria). Initially, linear models were fitted, and *p*-values were corrected for multiple testing using the Benjamini–Hochberg (BH) method to control the false discovery rate (FDR). The statistical significance of each gene was evaluated based on adjusted *p*-values (*p*adj), while log2 fold change (log2FC) values were used to assess differential gene expression between tumor and paired non-tumor samples.

The EMT pathway was evaluated using the Molecular Signatures Database (MSigDB) and Gene Set Enrichment Analysis (GSEA), with genes ranked according to log2FC values, using the clusterProfiler package (version 4.18.4) in the R environment. The correlation between HMGA2 expression and genes associated with the EMT pathway was assessed using Spearman’s correlation analysis. Overall survival analyses were performed after stratifying patients according to scores generated by single-sample Gene Set Enrichment Analysis (ssGSEA). The optimal cutoff point was determined using the survminer package (version 2.4.9). Survival curves were estimated using the Kaplan–Meier method, and differences between groups were assessed using the log-rank test.

### 4.11. Statistical Analysis

For in vitro assays, the statistical analyses were performed using GraphPad Prism software version 10.3.1 (GraphPad Software, Boston, MA, USA). For the functional assay results, a two-way ANOVA test was applied using mean and standard deviation (SD) values. For gene expression experiments, the Kolmogorov–Smirnov test was used to assess data normality, followed by the Mann–Whitney test, using data derived from three independent experiments. The significance threshold was set at *p* < 0.05. For the in silico analyses, expression data were transformed into log_2_ counts per million (log_2_CPM) to evaluate non-parametric correlations. Spearman’s correlation analysis was then performed to assess the association between HMGA2 expression levels and other genes of interest.

## Data Availability

The original contributions presented in this study are included in the article. Further inquiries can be directed to the corresponding author.

## References

[1] Bray F., Laversanne M., Sung H., Ferlay J., Siegel R.L., Soerjomataram I., Jemal A. (2024). Global Cancer Statistics 2022: GLOBOCAN Estimates of Incidence and Mortality Worldwide for 36 Cancers in 185 Countries. CA Cancer J. Clin..

[2] Yang H., Wang F., Hallemeier C.L., Lerut T., Fu J. (2024). Oesophageal Cancer. Lancet.

[3] Yang J., Liu X., Cao S., Dong X., Rao S., Cai K. (2020). Understanding Esophageal Cancer: The Challenges and Opportunities for the Next Decade. Front. Oncol..

[4] Li J., Xu J., Zheng Y., Gao Y., He S., Li H., Zou K., Li N., Tian J., Chen W. (2021). Esophageal Cancer: Epidemiology, Risk Factors and Screening. Chin. J. Cancer Res..

[5] Zhao Y.-X., Zhao H.-P., Zhao M.-Y., Yu Y., Qi X., Wang J.-H., Lv J. (2024). Latest Insights into the Global Epidemiological Features, Screening, Early Diagnosis and Prognosis Prediction of Esophageal Squamous Cell Carcinoma. World J. Gastroenterol..

[6] Zhang S., Shen Y., Zhu L., Lai Y., Zhu L., Xiao X., Li J., Tan W., Lin D., Wu C. (2026). Esophageal Cancer: From Pathogenesis to Precision Therapies. Signal Transduct. Target. Ther..

[7] Fusco A., Fedele M. (2007). Roles of HMGA Proteins in Cancer. Nat. Rev. Cancer.

[8] D’Angelo D., Mussnich P., Arra C., Battista S., Fusco A. (2017). Critical Role of HMGA Proteins in Cancer Cell Chemoresistance. J. Mol. Med..

[9] Jun K.-H., Jung J.-H., Choi H.-J., Shin E.-Y., Chin H.-M. (2015). HMGA1/HMGA2 Protein Expression and Prognostic Implications in Gastric Cancer. Int. J. Surg..

[10] Di Cello F., Hillion J., Hristov A., Wood L.J., Mukherjee M., Schuldenfrei A., Kowalski J., Bhattacharya R., Ashfaq R., Resar L.M.S. (2008). HMGA2 Participates in Transformation in Human Lung Cancer. Mol. Cancer Res..

[11] Sgarra R., Pegoraro S., Ros G., Penzo C., Chiefari E., Foti D., Brunetti A., Manfioletti G. (2018). High Mobility Group A (HMGA) Proteins: Molecular Instigators of Breast Cancer Onset and Progression. Biochim. Biophys. Acta (BBA) Rev. Cancer.

[12] Palumbo A., Da Costa N.M., Esposito F., De Martino M., D’Angelo D., De Sousa V.P.L., Martins I., Nasciutti L.E., Fusco A., Pinto L.F.R. (2016). HMGA2 Overexpression Plays a Critical Role in the Progression of Esophageal Squamous Carcinoma. Oncotarget.

[13] Kopka M.L., Yoon C., Goodsell D., Pjura P., Dickerson R.E. (1985). The Molecular Origin of DNA-Drug Specificity in Netropsin and Distamycin. Proc. Natl. Acad. Sci. USA.

[14] Miao Y., Cui T., Leng F., Wilson W.D. (2008). Inhibition of High-Mobility-Group A2 Protein Binding to DNA by Netropsin: A Biosensor-Surface Plasmon Resonance Assay. Anal. Biochem..

[15] Bartek J., Lukas C., Lukas J. (2004). Checking on DNA Damage in S Phase. Nat. Rev. Mol. Cell Biol..

[16] Satelli A., Li S. (2011). Vimentin in Cancer and Its Potential as a Molecular Target for Cancer Therapy. Cell. Mol. Life Sci..

[17] Ouchi K., Miyachi M., Yagyu S., Kikuchi K., Kuwahara Y., Tsuchiya K., Iehara T., Hosoi H. (2020). Oncogenic Role of HMGA2 in Fusion-Negative Rhabdomyosarcoma Cells. Cancer Cell Int..

[18] Xu X., Wang Y., Deng H., Liu C., Wu J., Lai M. (2018). HMGA2 Enhances 5-Fluorouracil Chemoresistance in Colorectal Cancer via the Dvl2/Wnt Pathway. Oncotarget.

[19] Chou T.-C., Talalay P. (1984). Quantitative Analysis of Dose-Effect Relationships: The Combined Effects of Multiple Drugs or Enzyme Inhibitors. Adv. Enzym. Regul..

[20] Huang F.-L., Yu S.-J. (2018). Esophageal Cancer: Risk Factors, Genetic Association, and Treatment. Asian J. Surg..

[21] Mansoori B., Mohammadi A., Ditzel H.J., Duijf P.H.G., Khaze V., Gjerstorff M.F., Baradaran B. (2021). HMGA2 as a Critical Regulator in Cancer Development. Genes.

[22] Chen Q., Fu Q., Pu L., Liu X., Liu Y. (2022). Effects of *HMGA2* Gene Silencing on Cell Cycle and Apoptosis in the Metastatic Renal Carcinoma Cell Line ACHN. J. Int. Med. Res..

[23] Tan L., Xu H., Chen G., Wei X., Yu B., Ye J., Xu L., Tan H. (2018). Silencing of HMGA2 Reverses Retardance of Cell Differentiation in Human Myeloid Leukaemia. Br. J. Cancer.

[24] Mori M., Ghirga F., Amato B., Secco L., Quaglio D., Romeo I., Gambirasi M., Bergamo A., Covaceuszach S., Sgarra R. (2023). Selection of Natural Compounds with HMGA-Interfering Activities and Cancer Cell Cytotoxicity. ACS Omega.

[25] Huso T.H., Resar L.M. (2014). The High Mobility Group A1 Molecular Switch: Turning on Cancer—Can We Turn It Off?. Expert Opin. Ther. Targets.

[26] Su L., Bryan N., Battista S., Freitas J., Garabedian A., D’Alessio F., Romano M., Falanga F., Fusco A., Kos L. (2020). Identification of HMGA2 Inhibitors by AlphaScreen-Based Ultra-High-Throughput Screening Assays. Sci. Rep..

[27] Pegoraro S., Ros G., Sgubin M., Petrosino S., Zambelli A., Sgarra R., Manfioletti G. (2020). Targeting the Intrinsically Disordered Architectural High Mobility Group A (HMGA) Oncoproteins in Breast Cancer: Learning from the Past to Design Future Strategies. Expert Opin. Ther. Targets.

[28] Meireles Da Costa N., Palumbo A., De Martino M., Fusco A., Ribeiro Pinto L.F., Nasciutti L.E. (2021). Interplay between HMGA and TP53 in Cell Cycle Control along Tumor Progression. Cell. Mol. Life Sci..

[29] Khazem F., Zetoune A.B. (2024). Decoding High Mobility Group A2 Protein Expression Regulation and Implications in Human Cancers. Discov. Oncol..

[30] Wu L., Wang Z., Lu R., Jiang W. (2012). Expression of High Mobility GroupA2 Is Associated with Poor Survival in Hepatocellular Carcinoma. Pathol. Oncol. Res..

[31] Zhao X.-P., Zhang H., Jiao J.-Y., Tang D.-X., Wu Y., Pan C.-B. (2016). Overexpression of HMGA2 Promotes Tongue Cancer Metastasis through EMT Pathway. J. Transl. Med..

[32] Rossini A., De Almeida Simão T., Marques C.B., Soares-Lima S.C., Herbster S., Rapozo D.C., Andreollo N.A., Ferreira M.A., El-Jaick K.B., Teixeira R. (2010). TP53 Mutation Profile of Esophageal Squamous Cell Carcinomas of Patients from Southeastern Brazil. Mutat. Res..

[33] Debnath P., Huirem R.S., Dutta P., Palchaudhuri S. (2022). Epithelial–Mesenchymal Transition and Its Transcription Factors. Biosci. Rep..

[34] Tan E.J., Thuault S., Caja L., Carletti T., Heldin C.-H., Moustakas A. (2012). Regulation of Transcription Factor Twist Expression by the DNA Architectural Protein High Mobility Group A2 during Epithelial-to-Mesenchymal Transition. J. Biol. Chem..

[35] Grant M.A., Baron R.M., Macias A.A., Layne M.D., Perrella M.A., Rigby A.C. (2009). Netropsin Improves Survival from Endotoxaemia by Disrupting HMGA1 Binding to the *NOS2* Promoter. Biochem. J..

[36] Pal A., Barrett T.F., Paolini R., Parikh A., Puram S.V. (2021). Partial EMT in Head and Neck Cancer Biology: A Spectrum Instead of a Switch. Oncogene.

[37] Aggarwal V., Montoya C.A., Donnenberg V.S., Sant S. (2021). Interplay between Tumor Microenvironment and Partial EMT as the Driver of Tumor Progression. iScience.

[38] Saitoh M. (2018). Involvement of Partial EMT in Cancer Progression. J. Biochem..

[39] Lüönd F., Sugiyama N., Bill R., Bornes L., Hager C., Tang F., Santacroce N., Beisel C., Ivanek R., Bürglin T. (2021). Distinct Contributions of Partial and Full EMT to Breast Cancer Malignancy. Dev. Cell.

[40] Peinado H., Ballestar E., Esteller M., Cano A. (2004). Snail Mediates E-Cadherin Repression by the Recruitment of the Sin3A/Histone Deacetylase 1 (HDAC1)/HDAC2 Complex. Mol. Cell. Biol..

[41] Gong J., Wang Y., Jiang B., Xu B., Hu C. (2019). Impact of High-Mobility-Group A2 Overexpression on Epithelial-Mesenchymal Transition in Pancreatic Cancer. Cancer Manag. Res..

[42] Wu H., Liang Y., Shen L., Shen L. (2016). MicroRNA-204 Modulates Colorectal Cancer Cell Sensitivity in Response to 5-Fluorouracil-Based Treatment by Targeting High Mobility Group Protein A2. Biol. Open.

[43] Martin J.C., Wartell R.M., O’Shea D.C. (1978). Conformational Features of Distamycin-DNA and Netropsin-DNA Complexes by Raman Spectroscopy. Proc. Natl. Acad. Sci. USA.

[44] Chirico V., Ergüven F.B., Möller K., Lutz F., Viehweger F., Kluth M., Hube-Magg C., Bernreuther C., Sauter G., Marx A.H. (2025). High Mobility Group Protein 2 (HMGA2) Is Highly Expressed in a Broad Range of Benign and Malignant Tumors. Virchows Arch..

[45] Ma Q., Ye S., Liu H., Zhao Y., Zhang W. (2024). The Emerging Role and Mechanism of HMGA2 in Breast Cancer. J. Cancer Res. Clin. Oncol..

